# Posterior Reversible Encephalopathy Syndrome as a Complication of Essential Thrombocythemia: A Case Report and Literature Review

**DOI:** 10.7759/cureus.82503

**Published:** 2025-04-18

**Authors:** Raul Hernandez-Iglesias, Pablo Arroyo-Pereiro, Alejandro Caravaca, Carla Marco, Maria Bea-Sintes, Alicia Senin, Jasson Andres Villareal-Hernandez, Monica Cos-Domingo, Antonio Martinez-Yelamos, Sergio Martinez-Yelamos, Albert Muñoz-Vendrell

**Affiliations:** 1 Department of Neurology, Hospital Universitari de Bellvitge, Barcelona, ESP; 2 Neurologic Diseases and Neurogenetics Group, Neuroscience Program, Institut d'Investigació Biomèdica de Bellvitge (IDIBELL), Barcelona, ESP; 3 Department of Clinical Hematology, Hospital Universitari de Bellvitge, Barcelona, ESP; 4 Department of Clinical Hematology, Hospital Duran i Reynalds - Institut Català d’Oncología, Barcelona, ESP; 5 Department of Clinical Hematology, Hospital Duran i Reynalds, Institut Català d’Oncología, Barcelona, ESP; 6 Department of Radiology, Institut de Diagnòstic per la Imatge (IDI) Universitari de Bellvitge (IDIBELL), Barcelona, ESP; 7 Department of Clinical Sciences, Facultat de Medicina, Universitat de Barcelona, Barcelona, ESP

**Keywords:** cortical blindness, essential thrombocythemia, posterior reversible encephalopathy syndrome (pres), refractory headache, vascular endothelial dysfunction

## Abstract

Posterior reversible encephalopathy syndrome (PRES) is a well-defined clinicoradiological syndrome that can arise in various clinical settings, most commonly in association with hypertensive states. However, less typical causes, including hematologic disorders, have also been reported.

A 47-year-old male with CALR-mutated essential thrombocythemia (ET) on chronic aspirin therapy developed progressive headache, drowsiness, nausea, and blindness. Workup revealed worsening thrombocytosis (1,250 × 10⁹/L) with unremarkable blood pressure, craniocervical AngioTac, and systemic assessment. PRES secondary to ET exacerbation was suspected. Hydroxyurea was initiated, leading to platelet reduction and symptom improvement. Brain MRI showed bilateral parieto-occipital Fluid-Attenuated Inversion Recovery (FLAIR) hyperintensities. At four months, only mild visual disturbances persisted, with follow-up MRI showing near-complete resolution.

ET decompensation may trigger PRES even with normal blood pressure, highlighting the importance of platelet monitoring to prevent complications. All reported cases involved middle-aged males with severe thrombocytosis (>700 × 10⁹/L) and symptom resolution following ET treatment and platelet reduction.

## Introduction

Posterior reversible encephalopathy syndrome (PRES) is an acute or subacute clinical-radiological entity characterized by a compatible presentation, a defined trigger, and reversible vasogenic edema on brain MRI. Although it can occur at any age, the mean onset is 45 years, with a slight female predominance [1]. Clinically, it presents with progressive headache, visual disturbances, altered consciousness, seizures, or coma [1-3]. A sudden or thunderclap headache may suggest reversible vasoconstriction syndrome (RVCS) instead [4]. MRI typically reveals bilateral, asymmetric parieto-occipital lesions, though other regions may also be affected, such as the frontal and temporal lobes, brainstem, cerebellum, and basal ganglia [2]. Diffusion-weighted imaging is the preferred modality for confirming the diagnosis of PRES, as it is crucial for distinguishing between vasogenic and cytotoxic edema [3].

The exact mechanism of PRES remains unclear, with two main theories proposed. The first attribute of PRES to a rapid rise in blood pressure, leading to a hypertensive emergency. This increase in cerebral perfusion pressure exceeds autoregulatory limits, causing endothelial dysfunction and plasma extravasation into the interstitium. Conversely, cerebral vasoconstriction in response to high perfusion pressure may induce ischemia [5]. The posterior brain is more vulnerable due to lower sympathetic innervation density compared to the anterior circulation [6]. The second theory explains PRES in normotensive patients, approximately 30% of cases, as a result of direct endothelial injury. Endogenous (preeclampsia, sepsis, autoimmune diseases) or exogenous (cytotoxic drugs) toxins activate the endothelium, increasing vascular permeability and promoting edema. In both mechanisms, blood-brain barrier disruption leads to abnormal fluid and protein leakage, resulting in vasogenic edema [3,5,6].

PRES has been linked to various conditions, particularly poorly controlled hypertension, renal failure, sepsis, neoplasms (solid and hematologic), autoimmune diseases (e.g., systemic lupus erythematosus, Crohn’s disease, rheumatoid arthritis), preeclampsia-eclampsia, cytotoxic drugs (e.g., cisplatin, gemcitabine, rituximab), and solid organ or bone marrow transplantation [1-3].

Essential thrombocythemia (ET), a very rare PRES-associated condition, has been reported in only two cases [7,8]. ET is a myeloproliferative disorder characterized by excessive megakaryocyte proliferation and sustained thrombocytosis (>450 × 10⁹/L for over one month) without secondary causes such as iron-deficiency anemia, malignancy, or infection [9,10]. The annual incidence of ET is 1.5-2.0 cases per 100,000 individuals. The median age of onset is between 60 and 70 years, although it can occur at any age. ET often presents as an asymptomatic incidental finding in otherwise healthy individuals, while others may present with headache, erythromelalgia, constitutional symptoms, or thrombotic and hemorrhagic events. Approximately 85-90% of patients have a mutation, the most common are either JAK2 or CALR [11]. Standard treatment includes daily 100 mg aspirin for thromboprophylaxis. Additional treatments are based on each patient's stratified risk; for example, patients over 60 with a history of prior ischemic events are typically candidates for cytoreductive therapy, often with hydroxyurea. Busulfan is reserved for elderly patients, while younger patients may receive interferon alpha and/or anagrelide [9].

This report aims to describe a rare case of PRES associated with ET and provide a comprehensive review of the existing literature.

## Case presentation

We presented a 47-year-old Caucasian male with well-controlled hypertension and JAK2-negative, CALR-mutated ET on chronic aspirin therapy (100 mg/day). Antiplatelet treatment was discontinued due to excessive thrombocytosis, and a bone biopsy was scheduled for further evaluation. Approximately one week later, he developed progressive drowsiness, a moderate frontal headache unresponsive to non-steroidal anti-inflammatory drugs (NSAIDs), nausea, and blurred vision. Within 24 hours, his symptoms worsened, presenting with severe headache, nausea, vomiting, bilateral blindness with positive visual phenomena (shapes and figures), and decreased consciousness. He had no fever, prior similar episodes, or other systemic symptoms.

Upon arrival at the emergency department, ophthalmologic evaluation revealed normal pupillary reflexes and fundoscopy, consistent with cortical blindness. Neurological examination confirmed bilateral blindness with only light perception, while the rest of the exam was unremarkable. Cranial CT and craniocervical angio-CT showed no parenchymal or vascular abnormalities. Blood pressure was 120/80 mmHg. Laboratory tests revealed thrombocytosis (1,250 × 10⁹/L) with preserved renal and hepatic function and normal red and white blood cell counts.

**Figure 1:**
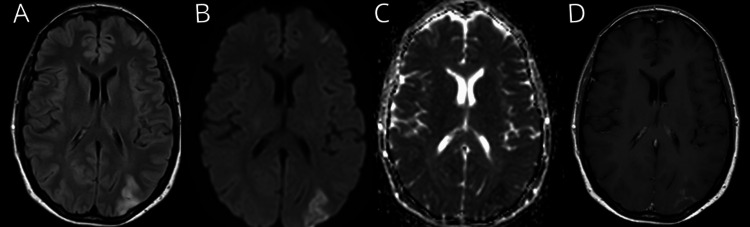
3T brain MRI, five days after symptoms onset. (A) FLAIR sequence shows bilateral parieto-occipital hyperintensities, predominantly in the left hemisphere. (B) DWI sequence shows bilateral posterior diffusion restriction. (C) ADC sequence demonstrates a reduced diffusion coefficient, predominantly in the left parieto-occipital region. (D) T1 sequence with gadolinium contrast reveals cortical-leptomeningeal enhancement in the left posterior parieto-occipital region. FLAIR, fluid-attenuated inversion recovery; DWI, diffusion-weighted imaging; ADC, apparent diffusion coefficient

A 3T brain MRI revealed Fluid-Attenuated Inversion Recovery (FLAIR) hyperintensity in the cortical-subcortical regions of the bilateral parieto-occipital lobes, predominantly on the left, with additional involvement of the left frontal region. There was diffusion restriction and patchy cortical-leptomeningeal contrast enhancement in the parieto-occipital area (Figure 1). Two serial electroencephalograms (EEGs), performed at different times during hospitalization to rule out seizures, showed no abnormalities. Further laboratory workup was negative for antinuclear antibodies, anti-ENA, anti-dsDNA, and antiphospholipid antibodies, as well as HIV, hepatitis, and syphilis serologies. Vitamin levels were normal, and blood protein electrophoresis showed no monoclonal bands.

Headache was managed with analgesics, and hydroxyurea (500 mg every 12 hours) was initiated for gradual platelet reduction, with a planned increase to 1,000 mg every 12 hours. Aspirin was not restarted due to bleeding risk, as severe thrombocytosis can lead to platelet dysfunction and acquired von Willebrand factor deficiency [10].

During the patient’s admission, he showed progressive clinical improvement, with headache resolution and enhanced visual acuity, allowing for reading. However, positive visual phenomena persisted as lights and shapes. He was discharged following platelet reduction and completion of the diagnostic workup.

**Figure 2:**
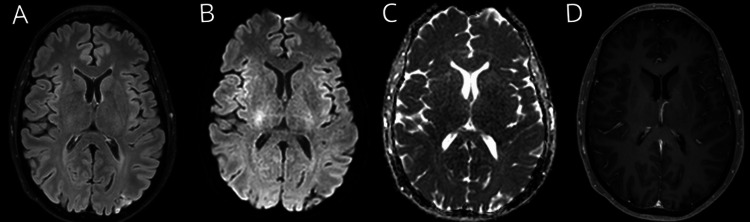
3T Brain MRI, four months later. (A) FLAIR sequence shows near-complete resolution of the left-predominant parieto-occipital hyperintensity. (B) DWI sequence shows absence of diffusion restriction. (C) ADC sequence shows absence of diffusion restriction. (D) T1 sequence with gadolinium contrast shows no contrast enhancement. FLAIR, fluid-attenuated inversion recovery; DWI, diffusion-weighted imaging; ADC, apparent diffusion coefficient

At four months, the patient remained in good general condition, headache-free, but with persistent mild visual hallucinations and a small central scotoma. Symptomatic neuromodulatory treatment was proposed for visual disturbances, but was declined by the patient. Antiplatelet therapy was resumed a few weeks post-discharge after the platelet count fell below 1,000 × 10⁹/L. Bone marrow biopsy confirmed myeloproliferative hematologic disease compatible with ET, with partial marrow fibrosis, not meeting myelofibrosis criteria. The patient remained asymptomatic, and no treatment modifications were necessary. [11,12]. Follow-up brain MRI showed near-complete resolution of prior abnormalities, confirming the reversibility of the condition (Figure 2).

**Figure 3:**
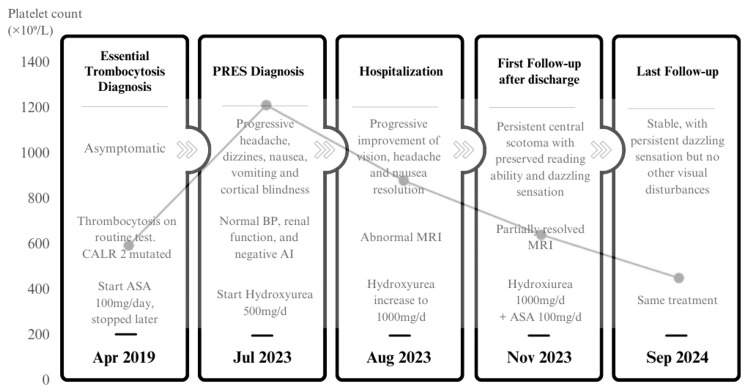
Patient evolution across different timepoints, illustrating platelet levels from ET diagnosis to PRES onset and follow-up in a line chart, along with clinical, diagnostic, and treatment data throughout the course. Image credits: Albert Muñoz-Vendrell and Raúl Hernández Iglesias. ET, essential thrombocythemia; PRES, posterior reversible encephalopathy syndrome; ASA, acetylsalicylic acid; BP, blood pressure; AI, autoimmunity; MRI, magnetic resonance imaging

Figure 3 illustrates the temporal evolution of ET and PRES, administered treatments, and platelet trends.

## Discussion

This case describes a patient who developed PRES following a documented exacerbation of ET with synchronous clinical improvement of both conditions, supporting a causal relationship.

In this patient, ET was diagnosed in April 2019 at the age of 43, following the incidental finding of thrombocytosis (600 × 10⁹/L) on a routine blood test, in the absence of specific symptoms. The patient remained asymptomatic for several years, receiving only low-dose aspirin (100 mg/day) as monotherapy.

The primary diagnostic suspicion, after excluding ophthalmologic causes of visual loss and acute posterior territorial stroke, was PRES. This diagnosis was supported by the clinical presentation, characterized by progressive headache, visual disturbances, and altered consciousness, along with MRI findings showing hyperintensity on FLAIR sequences, predominantly in the bilateral parieto-occipital regions with asymmetric predominance and contrast enhancement, which nearly resolved on follow-up imaging. Additionally, the likely precipitating factor was secondary thrombocytosis due to ET, occurring in the context of normal blood pressure.

Several potential differential diagnoses were considered and systematically excluded over time. Acute ischemic stroke was ruled out based on a normal initial CT angiography and brain MRI, which showed only minor involvement of the anterior cerebral artery territory. Cerebral venous thrombosis was excluded due to normal findings on venous imaging. Migraine with visual aura was considered unlikely given the persistence of visual symptoms lasting longer than one hour, in addition to pathological imaging findings. Reversible cerebral vasoconstriction syndrome (RCVS), which differs from PRES in its typical presentation with a thunderclap headache and is sometimes triggered by factors such as sexual activity, physical exertion, or certain medications, was also considered. RCVS diagnosis requires cerebral angiography demonstrating multifocal segmental vasoconstriction and a normal cerebrospinal fluid analysis [4]. However, in this case, neither angiography nor lumbar puncture was performed due to the patient's clinical improvement and the absence of strong clinical suspicion. Occipital seizures were ruled out by EEG, and central nervous system infections were excluded based on the absence of fever, inflammatory markers, and negative microbiological cultures.

To date, the association between PRES and ET has been reported in only two other cases [7,8]. Both involved middle-aged Asian males (49 and 60 years old) in whom PRES led to the diagnosis of ET. Symptom onset coincided with significant thrombocytosis (896 × 10⁹/L and 1,044 × 10⁹/L, respectively) and normal blood pressure. Similar to our case, autoimmune, metabolic, and serological studies were unremarkable, and MRI findings demonstrated the characteristic reversibility of PRES on follow-up imaging. A detailed summary of each of the three cases is presented in Table 1.

**Table 1:** Descriptive table of the clinical features of the two previously reported cases of PRES associated with ET, alongside our case. ET, essential thrombocythemia; PRES, posterior reversible encephalopathy syndrome; FLAIR, Fluid-Attenuated Inversion Recovery

Author	Zhang et al.	Li et al.	Hernández-Iglesias et al.
Year	2015	2020	2023
Age	49	60	47
Sex	Male	Male	Male
Medical History	None	High blood pressure and thrombocythemia	High blood pressure and ET
Chronic therapy	None	Aspirin 100 mg/day and telmisartan	Aspirin 100mg/d and losartan
Age at diagnosis of ET (years)	49	53	43
ET-associated mutation	JAK2 V617	DNMT3A W860R	CALR
Age at onset of PRES (years)	49	60	47
Time from ET diagnosis to PRES onset	At the same time	7 years	4 years
Time from aspirin discontinuation to PRES onset	Not applied	1 week	1 week
Clinical features	Headache, altered consciousness, and behavioral abnormalities	Progressive visual impairment and paroxysmal myoclonic movements	Headache, nausea, drowsiness, and blurred vision, which progressed to bilateral blindness
Platelet count at PRES onset	896 x 10E9/L	1,044 x 10E9/L	1,250 x 10E9/L
Cerebral MRI characteristics	Extensive vasogenic edema in the deep white matter of the right cerebellum and left occipital and temporal lobes	Restricted diffusion on DWI and reduced diffusion coefficient (ADC) in the bilateral parieto-occipital lobes	FLAIR hyperintensity in bilateral parieto-occipital lobes and left frontal region, with diffusion restriction and patchy contrast enhancement
Treatments	Aspirin, Mannitol and furosemide	Hydroxyurea and sodium bicarbonate	Hydroxyurea and symptomatic treatment
Platelet count control	465 x 10E9/L	620 x 10E9/L	450 x 10E9/L
Evolution	Completely symptomatic remission	Hallucinatory palinopsia until the fifth day, when it was completely recovered	Positive visual phenomena and a small central scotoma

The first case [7] involved a 49-year-old male with no significant medical history, who presented with headache, altered consciousness, and behavioral abnormalities. Initial MRI revealed extensive vasogenic edema in the deep white matter of the right cerebellum, as well as the left occipital and temporal lobes. The patient was treated with furosemide, mannitol, and supportive care for cerebral edema. Clinical improvement was observed within days, with follow-up MRI on day 9 demonstrating significant resolution of the initial lesions. Chronic aspirin therapy was initiated, and a third MRI on day 34 was normal. Serial platelet monitoring over the following months confirmed sustained normalization.

The second case [8] involved a 60-year-old male with a previously diagnosed thrombocytosis of unknown origin, for which he had been receiving chronic aspirin therapy (100 mg/day). Aspirin was discontinued one week before the onset of PRES. The patient presented with severe visual impairment, perceiving only light. Initial MRI revealed restricted diffusion on diffusion-weighted imaging (DWI) and a reduced diffusion coefficient on ADC in the bilateral parieto-occipital lobes. Treatment with hydroxyurea and supportive care resulted in rapid visual improvement, although hallucinatory palinopsia persisted until day 5. The patient also developed left upper limb tremors, resembling myoclonic seizures, during the first two days of illness, which subsequently resolved spontaneously. EEG showed periodic triphasic waves in the occipital region. Platelet levels gradually declined, and a follow-up MRI at four months confirmed complete resolution of PRES lesions. Importantly, both reported cases maintained normal blood pressure throughout PRES.

In ET, the most frequent driver mutations involve CALR, JAK2, or MPL, with CALR and JAK2 being the most common. In our case, a CALR mutation was detected, while JAK2 was negative. CALR mutations alter thrombopoietin receptor signaling, leading to sustained activation and excessive platelet production.

In our case, PRES onset occurred with a platelet count of 1,250 × 10⁹/L following the discontinuation of antiplatelet therapy. The subsequent clinical and radiological improvement with progressive platelet reduction suggests a relationship between thrombocytosis-induced endothelial dysfunction and the increased vascular permeability characteristic of PRES. Interestingly, the previously reported cases had different genetic profiles: one patient harbored the JAK2 V617F mutation, while the other had a DNMT3A W860R mutation [7,8].

Our case aligns with the endothelial dysfunction hypothesis of PRES, which occurs in approximately 30% of cases with normal blood pressure. Endothelial autoregulation maintains stable cerebral perfusion through vasodilators (e.g., nitric oxide) and vasoconstrictors (e.g., thromboxane A2 and endothelin) [13]. Patients with myeloproliferative (MP) disorders, such as polycythemia vera (PV) and ET, have an increased risk of thrombosis and hemorrhage. For example, myocardial infarctions without significant coronary stenosis are more common in these patients and are associated with endothelial dysfunction, which is driven by elevated vasoconstrictors, increased oxidative stress, and inhibition of the nitric oxide pathway, resulting in enhanced vasospasm [14]. Risk varies not only by disease group but also by mutation. For instance, patients with ET and the CALR mutation exhibit higher platelet counts, lower leukocyte and hemoglobin levels, and fewer thrombotic events compared to those with other mutations [15].

Notably, in both our patient and the second reported patient, PRES onset coincided with aspirin discontinuation one week prior [8]. Aspirin’s antithrombotic effect is well established and occurs through platelet inactivation via COX-1 inhibition, which prevents the synthesis of thromboxane A2, a potent vasoconstrictor and platelet activator [16]. Given that aspirin influences endothelial homeostasis by modulating prostaglandin and thromboxane synthesis, its withdrawal could contribute to a prothrombotic and proinflammatory state, exacerbating endothelial injury and increasing vascular permeability. This hypothesis is further supported by the follow-up MRI at four months, which showed near-complete resolution of PRES-related abnormalities after platelet normalization, reinforcing both the reversible nature of PRES and the potential role of platelet-driven endothelial dysfunction in its pathogenesis. In fact, in conditions with pathophysiology similar to PRES, such as in women at high risk of preeclampsia, low-dose aspirin has been demonstrated to be the most effective preventive measure [17].

This report has several limitations. The validity of a single case report of PRES associated with ET is limited; however, due to the rarity of such cases, it contributes valuable insights to the field. No lumbar puncture or invasive angiography was performed; nevertheless, the temporal sequence and synchronous improvement of both conditions were deemed sufficient to establish the diagnosis and exclude other potential causes. Further reports involving a larger number of cases would be necessary to better understand the relationship between clinical phenotypes, chronic treatment or its discontinuation, various mutations, and the risk of PRES in these patients.

## Conclusions

In conclusion, this case, along with previously reported ones, suggests that ET should be considered a potential cause of PRES in appropriate clinical settings.

In clinically similar cases, it is crucial to exclude ophthalmological pathology, ischemic stroke, cerebral venous thrombosis, and occipital epilepsy, and consider other conditions such as migraine with aura, reversible cerebral vasoconstriction syndrome, autoimmune diseases like antiphospholipid syndrome and lupus, as well as infections. If these conditions are excluded, along with compatible imaging findings, a diagnosis of PRES related to ET may be suspected.

Furthermore, we recommend that patients with ET undergo careful monitoring of platelet levels, as significant elevations can lead to complications such as PRES, particularly in cases where oral aspirin therapy must be discontinued for any reason.
